# Original Antigenic Sin: the Downside of Immunological Memory and Implications for COVID-19

**DOI:** 10.1128/mSphere.00056-21

**Published:** 2021-03-10

**Authors:** Eric L. Brown, Heather T. Essigmann

**Affiliations:** a Center for Infectious Disease, Division of Epidemiology, Human Genetics, and Environmental Sciences, University of Texas Health Science Center, Houston, Texas, USA; University of Florida

**Keywords:** B cells, COVID-19, germinal center, affinity maturation, germinal center reaction, humoral immunity, immunity, imprinting, memory responses, original antigenic sin, vaccines

## Abstract

The concept of original antigenic sin (OAS) was put forth many years ago to explain how humoral memory responses generated against one set of antigens can affect the nature of antibody responses elicited to challenge infections or vaccinations containing a similar but not identical array of antigens. Here, we highlight the link between OAS and the germinal center reaction (GCR), a process unique to activated B cells undergoing somatic hypermutation and class switch recombination. It is the powerful response of activated memory B cells and the accompanying GCR that establish the foundations of OAS. We apply these concepts to the current COVID-19 pandemic and put forth several possible scenarios whereby OAS may result in either beneficial or harmful outcomes depending, hypothetically, on prior exposure to antigens shared between SARS-CoV-2 and seasonal human coronaviruses (hCoVs) that include betacoronaviruses (e.g., HCoV-OC43 and HCoV-HKU1) and alphacoronaviruses (e.g., HCoV-NL63 and HCoV-HKU1) (E. M. Anderson, E. C. Goodwin, A. Verma, C. P. Arevalo, et al., medRxiv, 2020, https://doi.org/10.1101/2020.11.06.20227215; S. M. Kissler, C. Tedijanto, E. Goldstein, Y. H. Grad, and M. Lipsitch, Science 368:860–868, 2020, https://doi.org/10.1126/science.abb5793).

## OPINION/HYPOTHESIS

The speed and specificity of immunological memory are the basis of long-term, acquired immunity and of vaccinology. The power of memory, however, comes at a price. The cost? A memory response triggered to a similar but not identical array of antigens (e.g., a new exposure to a related but antigenically distant pathogen) can potentially be less effective than a response elicited in the absence of memory (1–4). This is possible because memory B cells producing antibodies of high affinity and specificity established following a primary exposure to one subset of antigens can prevent or significantly dampen responses by naive B cells to new antigens if they are part of a profile that includes antigens present during the primary exposure (5, 6). This is not a problem if the memory response produces neutralizing antibodies to antigens associated with the secondary exposure; however, problems do arise if memory B cells produce nonneutralizing antibodies to the antigens shared between primary and secondary exposures as reported recently in humans exposed to related human coronaviruses (hCoVs) and later infected with SARS-CoV-2 (7, 8). In such a scenario, not only can the memory response be ineffective, it can significantly attenuate the response of newly activated B cells that could have responded effectively to new antigens absent from the original priming event. The overwhelming response of memory B cells to cognate antigens that can hinder naive B cells of different and possibly neutralizing specificities from effectively responding to a new stimulus is known as the original antigenic sin (OAS), a biblical reference suggesting that the immune system is bound by the “sin” of its first imprinting to a target (1, 2). For example, people infected with H1N1 influenza viruses during childhood (and thus imprinted with that set of antigens/epitopes) were protected later in life against infections with a related virus such as H5N1 but not infections with more distantly related H3N2 (3, 9, 10).

Recently, a hypothesis referred to as “antigenic seniority” has been proposed as an alternative to OAS (11). Antigenic seniority explains dominant antibody responses as a consequence of repeat exposures to the same antigen(s) rather than to the first antigenic exposure or imprinting that is the core tenet of OAS (6). These differences, however, are not important to the present discussion as both OAS and antigenic seniority are bound by their common denominator: the germinal center reaction (GCR). Here, we discuss the immunological foundations of OAS, particularly with respect to the generation and subsequent selection of high-affinity B cells during GCRs and how these processes could be beneficial or harmful in the context of COVID-19, keeping in mind that without the power of immunological memory OAS would not exist.

## WHAT MAKES THE GENERATION OF B CELL AND T CELL RESPONSES DIFFERENT? THE GERMINAL CENTER REACTION (GCR)

It’s important to note that early development of B cells and T cells, the pillars of acquired immunity, follow parallel paths (12). Both cell types derive from a common pluripotent progenitor cell, and after “choosing” their respective developmental paths, both rearrange genes to produce either antigen-specific B cell receptors (BCRs or antibodies in their secreted form; recognizing three-dimensional structures) or T cell receptors (TCRs; recognizing peptides in the context of major histocompatibility molecules) (12). Furthermore, both cell types undergo similar selection processes to ensure that most cells emerging from the bone marrow (B cells) and the thymus (T cells) are primarily reactive to yet-to-be-encountered, pathogen-derived antigens (13–17). The similarities end there.

First, naive T cells that emerge from the thymus are present for life, so thymectomy after puberty does not significantly affect a person’s T cell repertoire (12). In contrast, most naive B cells emerging from the bone marrow each day do not survive in the periphery, and those that do will survive for only a short period if they do not encounter their cognate antigen (12, 18). The B cell repertoire is therefore continually refreshed. Second and most relevant to OAS, T cells successfully emerging from the rigorous thymic selection process never again have to survive a second rearranging of their TCR genes (12).

In contrast, naive B cells activated following ligation of their cognate antigens via the BCR (and further activated by signals received by helper T cells) enter the GCR to undergo affinity maturation, a process designed to increase activated B cells’ affinity to their cognate antigens (6, 19). Here, B cells undergo repeated rounds of somatic hypermutation, driven by the enzyme activation-induced cytidine deaminase (AID), which introduces mutations at cytidine hot spots located primarily within the variable domain gene sequences of the BCR in hopes of introducing favorable mutations to increase the affinity of the antibody to its activating antigen (6, 20). After each attempt at somatic hypermutation, B cells within the GCR compete against each other, and only those clones that repeatedly acquire favorable mutations to their BCR will survive multiple rounds through the GCR gauntlet (19). In the end, only the few select survivors of this harrowing Darwinian selection process will emerge to produce high-affinity antibodies. Following any subsequent exposure(s) to their cognate antigen, these B cells will reactivate and respond rapidly but oftentimes not before surviving a new GCR and further rounds of selection (19), resulting in a new subset of B cells expressing BCRs of even higher affinity than before. Thus, the affinity of BCRs to specific antigens changes following postprimary exposures to the same antigen while the affinity of a T cell clone to its cognate antigen never does. This is observable in the increased number of mutations, compared to that of the germ line sequence, within variable domain sequences of BCR genes following repeated rounds of vaccination or infection (12, 20).

## THE DOWNSIDE OF MEMORY: THE ORIGINS OF ORIGINAL ANTIGENIC SIN (OAS)

Memory is never cast as a “bad guy”; after all, it is the power of immunological memory that keeps us safe from reinfection with the same pathogen if neutralizing immunity is elicited following primary exposure or vaccination. The problems with OAS begin following exposure to a related strain of a pathogen that possesses a similar but not identical panel of antigens (i.e., greater antigenic distance/difference) (3). It is important to remember that OAS cannot occur without the GCR: OAS exists because B cells surviving the GCR express BCRs of such high affinity that naive B cells with specificities to new antigens receiving activating signals via their BCR for the first time will stand little chance of competing successfully against seasoned memory B cells that activate at lower signaling thresholds following reexposure to their cognate antigen, independent of the fact that the memory and naive B cells in this scenario likely have BCRs with different specificities (4, 19). Therefore, if memory B cells respond to an infection with a related but antigenically distant pathogen, the memory response can not only be ineffective but possibly diminish the effectiveness of naive B cells capable of producing neutralizing antibodies (5). This potential downside of memory must be considered when attempting to design vaccines lest a vaccine formulation induce an immunological setback that precludes the elicitation of protective immunity or to understand the production of protective or nonprotective immunity to a new strain of a pathogen. This scenario recently played out following the release of the human papillomavirus (HPV) vaccine Gardasil 9 that contains four antigens present in the original Gardasil vaccine plus an additional five new antigens. Individuals previously immunized with Gardasil who were later vaccinated with Gardasil 9 mounted poor responses to the five new antigens present in the Gardasil 9 vaccine compared to individuals vaccinated with Gardasil 9 who had no prior exposure to Gardasil (21).

In the context of vaccine design, the antigenic distance hypothesis was put forth to explain how differences in vaccine efficacy were impacted by the distance or relatedness of prior vaccine strains (22). That is, if the antigenic distance of respective vaccines is greater than that of circulating virus strains, efficacy is compromised (3, 22). A recent example occurred during the 2014–2015 influenza season when a new glycosylation site acquired by the circulating H3N2 strain was absent from the vaccine strains (3). Adults previously infected during childhood with influenza strains deficient in this glycosylation elicited strong responses against the vaccine strain but were poorly equipped immunologically to prevent infections with the glycosylated 2014–2015 H3N2 isolate (23–25).

## ROLE OF OAS IN SARS-CoV-2 INFECTION AND IMMUNITY

**FIG 1:**
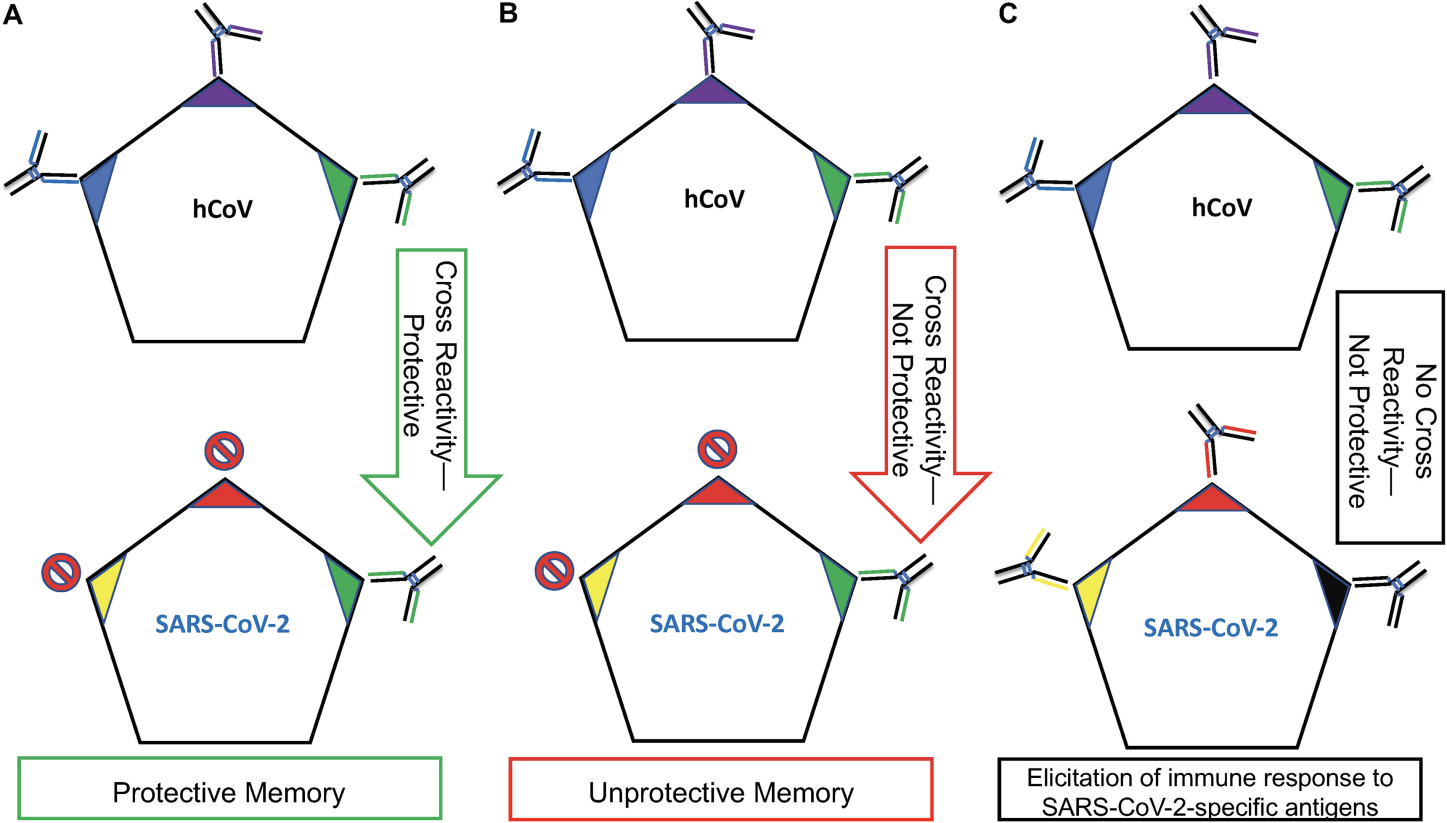
The impact of OAS on the efficacy of the immune response. OAS is affected not only by the temporality of an antigenic exposure but also by the “collection” of antigens associated with the exposure. (A) Exposure to hCoVs (related viruses such as HCoV-OC43, HCoV-HKU1, HCoV-NL63, and HCoV-HKU1) primes the immune system to the Blue, Purple, and Green antigens. This will result in long-lived memory B cells with specificities to these antigens, respectively. Reexposure to hCoVs will result in a robust antibody response to each of these antigens. SARS-CoV-2 expresses the same hCoV Green antigen and two new antigens, Yellow and Red. The memory anti-Green B cell response will prevent or significantly limit the ability of naive B cells with specificities to Yellow and Red antigens from developing. In this scenario, the anti-Green antibody is protective; so, while OAS prevented/diminished the elicitation of antibodies with specificities to the Yellow and Red antigens, the anti-Green antigen memory response confers some level of protection against a SARS-CoV-2 infection. (B) This scenario is identical to that described for panel A with the exception that the anti-Green response elicited against hCoV is nonneutralizing for SARS-CoV-2. In this example, the memory response to Green is nonprotective while simultaneously inhibiting/interfering with the ability to mount a new response to the Yellow and Red antigens that could potentially provide protection. (C) This scenario depicts two separate exposures. Since no antigens are shared between hCoVs and SARS-CoV-2, the anti-SARS-CoV-2 response will be a primary exposure, unaffected positively or negatively by prior exposure to hCoVs.

Figure 1 illustrates three hypothetical scenarios that could play out following a SARS-CoV-2 infection in the context of a prior exposure to a related hCoV. First exposure to hCoVs (e.g., betacoronaviruses such as HCoV-OC43 and HCoV-HKU1 or alphacoronaviruses HCoV-NL63 and HCoV-HKU1 shown to possess degrees of antigenic similarity) will result in the activation of B cell clones reactive, respectively, to the Blue, Purple, and Green antigens unique to hCoVs (26). Each of these B cell clones will have been activated simultaneously and survived their respective GCRs, resulting in three distinct memory B cell lineages.

The best-case scenario involves previous exposure to hCoVs sharing a protective epitope (Green) identical to or very similar to that expressed by SARS-CoV-2 (Fig. 1A). The anti-Green response elicited here by a respective hCoV confers some level of protection because anti-Green antibodies are neutralizing against a SARS-CoV-2 infection. For example, antibodies generated against a conserved domain, such as the spike glycoprotein S2 subunit, could confer cross-protective immunity across hCoVs and SARS-CoV-2 (26). In this context, Ng et al. (26) demonstrated a scenario similar to that described in Fig. 1A whereby SARS-CoV-2 was neutralized *in vitro* using antibodies present in the serum of SARS-CoV-2-uninfected individuals previously exposed to hCoVs (21). Because of OAS, no significant responses will likely be generated against the SARS-CoV-2 Red and Yellow antigens since the anti-Green memory response is so overwhelming that elicitation of a natural anti-Red and anti-Yellow response is prevented or significantly diminished. For the purposes of this discussion, it does not matter if the Red or Yellow antigens are conserved or not, nor does it matter if antibodies generated to the Red or Yellow antigens are neutralizing or not since the tenet of Fig. 1A is the dominant and overwhelming memory/GCR response to the Green antigen. As shown here, for OAS to be of benefit exposure to antigens eliciting protective immunity must be the same or similar between primary and secondary exposures and beyond (3). Had exposure to the Red and Yellow antigens occurred in the absence of the Green antigen, an immune response spearheaded by naive B cells unimpeded by an anti-Green memory B cell response would enter the GCR followed by the establishment of anti-Red and -Yellow memory.

The scenario depicted in Fig. 1B represents a worst case in the context of immunity against a SARS-CoV-2 infection for two reasons: (i) the antibodies produced by memory B cells reactive to the SARS-CoV-2 Green antigen are not protective, and (ii) the strength of the memory response stimulated by the Green antigen may hinder the elicitation of potentially neutralizing antibodies to SARS-CoV-2-specific Red and Yellow antigens. This scenario is of particular concern if a potentially protective response to the receptor binding domain (RBD) of SARS-CoV-2 (e.g., the Red or Yellow antigens in Fig. 1B) is dampened as a result of OAS (27). Furthermore, so long as the unprotective Green antigen is present, neutralizing immunity is unlikely to develop over time following natural exposure to SARS-CoV-2. Anderson et al. described this scenario by demonstrating that SARS-CoV-2 cross-reactive but nonneutralizing antibodies were present in approximately 20% of people exposed to hCoVs prior to the start of the SARS-CoV-2 outbreak (16.2% had antibodies to SARS-CoV-2-N protein and 4.2% to the SARS-CoV-2-S protein) (7). This raises a question: is there a Green antigen equivalent in some circulating hCoVs that not only elicits the production of nonneutralizing/nonprotective antibodies but also, as a consequence of OAS, hinders elicitation of protective antibody responses following a SARS-CoV-2 infection?

The presence of nonneutralizing but cross-reactive antibodies to SARS-CoV-2 antigens in prepandemic serum samples is a reminder that detection of an antibody signature does not equate to protection against infection. One need only look at cross-protective immunity, or lack thereof, in the context of dengue virus infections. Infection with one of the four dengue serotypes elicits a neutralizing antibody response to that serotype only (28). Such antibodies not only are generally nonneutralizing to the other three serotypes but can also worsen outcomes by accelerating dengue virus uptake into human cells via the mechanism of antibody-dependent enhancement (ADE). Although OAS plays a role in ADE, currently it does not appear to play a role in facilitating SARS-CoV-2 uptake. We therefore use ADE as a reminder that not all antibody responses are helpful and some can be harmful (29–31).

The scenario depicted in Fig. 1C does not involve memory. In this scenario, no antigens are shared between hCoVs and SARS-CoV-2. This means that immunity to SARS-CoV-2 would develop unimpeded and unaided by OAS, hopefully resulting in the elicitation of neutralizing antibody responses to SARS-CoV-2-specific antigens over time.

## CONSIDERATION OF OAS FOR VACCINE DEVELOPMENT

Future studies across populations and age groups will determine the impact of OAS in the context of COVID-19. Recent data suggest that the scenarios described in Fig. 1A and B are possible in the context of SARS-CoV-2 and prior exposures with hCoVs (7, 8, 32, 33).

The impact of OAS on the elicitation of protective immunity should not be ignored in vaccine development. Selection of a vaccine candidate or candidates that are too similar to antigens already “seen” by the population at large could result in three distinct outcomes: (i) a “back-boost” or enhanced protective immunity resulting from a second round of GCRs in response to shared antigens between primary and secondary exposures (Fig. 1A) (34), (ii) boosting of a nonprotective antibody response (Fig. 1B), or (iii) in the context of a multicomponent vaccine formulation, the masking of a protective response against some vaccine components if other antigens in the formulation have been previously “seen” by the population as observed with Gardasil 9 (Fig. 1B) (21).

OAS is the double-edged sword of memory: it can provide an avenue to protection against a novel strain of a pathogen or create an obstacle to the elicitation of protective immunity. What this means for COVID-19 is yet to be fully determined, but this important consequence of the immense powers of immunological memory and specificity must be considered when assessing population-level immunity and vaccine efficacy.
